# Effect of neurodevelopmental treatment on gross motor function and daily living in children with cerebral palsy

**DOI:** 10.4102/ajod.v14i0.1758

**Published:** 2025-10-15

**Authors:** Aditya D. Pratama, Haunan N. Izdihar, Marilyn Moffat

**Affiliations:** 1 Department of Physiotherapy, Faculty of Vocational Education Program, University of Indonesia, Depok, Indonesia; 2 Department of Physical Therapy, Graduate School of Education, New York University, New York, United States; 3 Department of Physical Therapy, Institute of Health Professions, Massachusetts General Hospital, Boston, United States; 4 Department of Health and Physiology, Graduate School of Arts and Science, New York University, New York, United States

**Keywords:** neurodevelopmental treatment, cerebral palsy, gross motor function, activities of daily living, spastic diplegia, rehabilitation, physiotherapy, Indonesia

## Abstract

**Background:**

Daily tasks can be challenging for young individuals with motor impairments caused by brain disorders. Neurodevelopmental treatment (NDT) aims to enhance motor function by concentrating on the central nerve and neuromuscular systems. However, research on the effectiveness of NDT for children with spastic diplegia in Indonesia, particularly regarding gross motor skills and daily activities, is limited.

**Objectives:**

This study aimed to investigate the effects of NDT on activities of daily living (ADL) and gross motor skills in children with spastic diplegia.

**Methods:**

This study utilised a pre-test–post-test experimental design. Twenty children diagnosed with spastic diplegia were recruited via purposive sampling from a specialised paediatric rehabilitation centre. The Modified WeeFIM was used to assess ADL, and the GMFM-88 was utilised to measure gross motor abilities. The NDT intervention was administered over the course of 8 weeks, twice a week.

**Results:**

Activities of daily living (*p* < 0.001, mean improvement of 13.6) and gross motor abilities (all GMFM-88 tests, *p* < 0.05) showed significant improvements. A substantial favourable association (*r* = 0.702; *p* < 0.001) was observed between GMFM-88 and WeeFIM scores, suggesting that improvements in motor skills were associated with better daily functioning.

**Conclusion:**

Neurodevelopmental treatment helped children with spastic diplegia with their everyday activities and motor function, supporting its role in promoting independence.

**Contribution:**

This study fills a gap in research by evaluating NDT’s impact on daily activities and Indonesian children with cerebral palsy and their motor function, contributing to a deeper understanding of its benefits.

## Introduction

The most frequent reason why children have long-term physical disabilities, namely cerebral palsy (CP), affects 17 million individuals globally and is present in one out of every 500 infants (Khan et al. 2022). According to Upadhyay et al. (2020), CP is a collection of non-progressive, irreversible problems of movement and posture development that are caused by abnormalities in the growing foetus or newborn brain. Along with epilepsy and secondary musculoskeletal issues, these main motor abnormalities are frequently accompanied by related deficits in sensation, cognition, communication and behaviour (Upadhyay et al. 2020). Motor impairments in CP occur because of brain damage that affects the child’s coordination, balance and movement patterns (Upadhyay, Tiwari & Ansari 2020).

Being born prematurely and having a low birthweight are among the primary factors that elevate a newborn’s risk of developing CP. Additional variables that raise the chance of CP include multiple pregnancies, certain genetic conditions (such as specific gene mutations), maternal infections passed to the infant, lack of oxygen to the brain, perinatal stroke, neonatal seizures and severe infections like meningitis (Pavone et al. 2021).

The prevalence of CP shows substantial variation across different countries and regions, influenced largely by economic status. In high-income countries, approximately 1.5 to 3 out of every 1000 live births are affected by CP (Patel et al. 2020; Sadowska et al. 2020). In contrast, estimates in Indonesia suggest that between one and five children per 1000 live births are impacted by this condition (Salfi, Sahorso & Atika 2019; Das & Ganesh 2019). As of 2023, West Java Province reported the highest number of children with developmental delays in the country, totalling 154 476 cases. Within this context, CP was reported to have a prevalence rate of 0.1%, a figure at the lower end of national estimates (BKPK 2023).

Spastic diplegia, a common subtype of CP, primarily affects motor control in the legs more than the arms and is often characterised by central hypotonia and related muscle weakness in the trunk (Abbas et al. 2024). This lack of trunk control is a primary reason for the challenges with gross motor abilities, including movement and walking, commonly observed in these children (Wang et al. 2024).

These motor skill deficits have cascading consequences on a child’s daily life, which is comprehensible using the International Classification of Functioning, Disability, and Health (ICF) framework (Karmomyanan et al. 2023). Delays in the development of gross motor skills (an impairment of body function) are closely linked to limitations in performing daily functional tasks (activity limitations) (Emara 2015). Ultimately, these limitations restrict a child’s involvement in social, educational and community roles, an issue defined as participation restriction (Khanna et al. 2023). Such severe restrictions in participation can negatively impact their health-related quality of life (Lucas et al. 2016), as underlying movement impairments make it harder for them to perform everyday tasks independently (Karmomyanan et al. 2023).

Neurodevelopmental therapy (NDT), developed by Berta and Karel Bobath, has been a widely recognised treatment modality for children with CP since the 1940s (Tekin et al. 2018). This therapeutic approach is a problem-solving strategy that targets motor and postural abnormalities resulting from lesions inside the central nervous system (Primadasa & Widodo 2022). The core of NDT involves direct therapeutic handling to inhibit atypical movement patterns while facilitating more typical ones. Therapists use specific ‘key points of control’ on the trunk and limbs to influence muscle tone and guide the child through functional activities like sitting, reaching or transitioning between positions. Targeting the neuromuscular and central neural systems, this therapy aims to maximise functional independence and improve gross motor efficiency (Sah, Balaji & Agrahara 2018). The ultimate goal is the habituation of these new motor patterns, where the child learns to automatically incorporate them into daily routines, leading to improved overall function and participation.

Based on existing evidence, NDT appears to contribute to enhancing gross motor performance and everyday capabilities among paediatric patients with CP (Novak et al. 2020). However, more focused research is required. To yield specific findings, this study concentrated on youngsters who suffer from spastic diplegia, a common form of CP whose characteristic impairments in trunk control and lower limb function are key targets for the NDT approach. For children with CP in Indonesia, this study aims to thoroughly explore the impact of NDT on both gross motor skills and independence in daily activities (e.g. self-care, mobility). It is hypothesised that NDT has a significant, positive impact on these outcomes. Furthermore, the study will assess if an association exists between gains in gross motor function and improvements in daily activity performance following the intervention.

## Research methods and design

### Sample size

This quantitative study utilised a pre-experimental, pre-test-post-test design conducted without a control group (Adiputra et al. 2021). It took place from February 2025 to March 2025 at a private, specialised paediatric rehabilitation centre located in a major urban area in Indonesia. A total of 20 participants were recruited via purposive sampling based on detailed eligibility criteria. Inclusion criteria for participants were as follows: (1) A spastic diplegia diagnosis verified by a paediatric neurologist at least 1 year prior to the study; (2) an age range of 5–12 years. This range was selected as children in this developmental stage are typically able to participate actively in therapy and assessments, while their motor patterns are still adaptable to intervention; and (3) children with CP who are actively undergoing therapy at Ramah Cerebral Palsy (RCP) Bogor. All participants were actively undergoing regular therapy at the centre. Children were excluded if they had undergone orthopaedic surgery during the previous 12 months or had received injections of botulinum toxin, which could confound the results. Prior to participation, the parents or legal guardians of every child provided written, informed consent.

### Outcome measures

Two standardised instruments were employed to evaluate therapy outcomes: ‘GMFM-88’ and ‘WeeFIM’.

The GMFM-88 is a criterion-referenced test designed to measure motor function in children with CP. Walking, sprinting, leaping, crawling, kneeling, sitting, rolling over and standing are the five domains into which its 88 tasks are divided. The GMFM-88 is acknowledged for its good construct validity for this demographic and its strong test-retest and inter-rater reliability (Intra-class Correlation Coefficient [ICC] > 0.98), providing statistical justification for its use (Alotaibi et al. 2013; Anggoro et al. 2012).

To evaluate functional independence in daily activities, this study utilised a 25-item Modified WeeFIM. This specific version was previously adapted and validated for use in children with developmental disorders in Indonesia (Kim et al. 2022). The instrument assesses two domains: self-care and mobility, with item scores ranging from 1 (completely dependent) to 5 (fully independent). In its validation study, this modified instrument demonstrated high test-retest reliability (ICC = 0.89), as well as internal consistency (Cronbach’s alpha = 0.92) (Kim et al. 2022). This established psychometric evidence ensures its appropriateness and justifies its application in this study.

### Intervention

The intervention was delivered by two certified Neurodevelopmental Treatment (NDT) physiotherapists, each possessing more than 5 years of clinical experience in paediatric rehabilitation. All participants attended 60-min individual therapy sessions twice weekly over an 8-week period, for a total of 16 sessions. The physiotherapy programme was based on NDT principles and individually designed to meet the unique requirements of children suffering from spastic diplegia, focusing on improving trunk control, modulating muscle tone and facilitating more typical movement patterns. Key treatment strategies involved the facilitation of postural control by utilising therapeutic balls and bolsters to activate core musculature; the inhibition of atypical tone through specific handling techniques like slow, rhythmic rotation of the trunk over the pelvis; and the guidance of functional movements such as sit-to-stand using key points of control to ensure proper alignment and weight-bearing. These techniques were integrated into task-oriented practice, such as reaching for objects or practising stepping patterns, to enhance motor learning and functional carryover into daily life.

### Research procedure

At the start of the study, parents or guardians provided written consent and participated in interviews to gather basic demographic information, including the child’s identity, age range and sex classification. During the week prior to the intervention, pre-assessments were conducted by the two certified NDT physiotherapists involved in the study. The Modified WeeFIM was administered via structured interviews with parents or guardians, and the GMFM-88 was assessed by seeing the child’s performance up close. After the 8-week intervention, post-intervention assessments using the same instruments and procedures were administered to evaluate changes in motor and functional performance.

### Statistical analysis

SPSS (Version 26.0) software was used for statistical analysis. To ascertain if the data distribution was normal, the Shapiro-Wilk test was employed. Respondent characteristics were described using descriptive statistics; measures of central tendency were presented as the median for data that are not normally distributed and the mean and standard deviation for data that are normally distributed. The selection of inferential statistical tests was based on the outcome of the normality test. The data for the Modified WeeFIM scores were found to be normally distributed; therefore, the pre- and post-intervention scores were suitably compared using the paired t-test. Conversely, the GMFM-88 scores were not normally distributed, which necessitated using the Wilcoxon signed-rank test, which is non-parametric. To look at the relationship between gross motor skills (GMFM-88 scores) and daily activities (Modified WeeFIM scores), the correlation between Spearman’s rank and order was employed. This test is non-parametric and was chosen because the GMFM-88 data did not meet the assumption of normality required for a Pearson correlation test.

### Ethical considerations

This study involving human subjects was carried out in compliance with national regulations and institutional policies, following the Declaration of Helsinki’s ethical guidelines (World Medical Association 2013). This study was approved ethically by the Ethics Committee of the Vocational Education Programme, Universitas Indonesia, on 14 February 2025 (Approval No.: KES-53/01/2025).

## Results

### Participant characteristics

Table 1 presents a summary of participants’ characteristics, including age, sex and their scores on the GMFM-88 and Modified WeeFIM assessments, both before and after the intervention.

**TABLE 1:** Demographic and baseline characteristics of participants.

Age (years)	Frequency	Valid percent
5	8	40.0
6	2	10.0
7	2	10.0
9	1	5.0
11	3	15.0
12	4	20.0

**Total**	**20**	**100.0**

Note: Mean = 7.80; Min-Max = 5–12.

The age distribution revealed that most participants (40%) were 5 years old. Other age groups, such as 6 years, 7 years and 12 years old, represented between 10% and 20% of the total sample. Although the age range varied, children aged 5 made up the largest group. The composition of participants showed a difference in the proportion of responders who were male and female.

Among the 20 participants, 11 were male (55%) and 9 were female (45%). The results of the pre-test and post-test for (GMFM-88) and (Modified WeeFIM) showed an increase across all dimensions, demonstrating an overall improvement. In GMFM-88, the average scores across all dimensions increased from pre-test to post-test. Similarly, the average Modified WeeFIM score rose from 67.1 during the pre-test to 80.7 in the post-test. Tables 2 and 3 summarise the pre- and post-intervention scores for GMFM-88 and WeeFIM.

**TABLE 2:** Pre-test and post-test distribution of GMFM-88.

GMFM	Mean	Median
Pre_Dim A	46.25	47.0
Post_Dim A	51.00	51.0
Pre_Dim B	47.90	50.5
Post_Dim B	54.35	57.5
Pre_Dim C	18.50	12.5
Post_Dim C	23.45	28.5
Pre_Dim D	5.65	0.0
Post_Dim D	9.20	3.5
Pre_Dim E	6.75	0.0
Post_Dim E	10.10	3.0

GMFM, Gross Motor Function Measure; Dim, dimension.

Note: Key for dimensions: Dim A: Lying and rolling, Dim B: Sitting, Dim C: Crawling and kneeling, Dim D: Standing, Dim E: Walking, running and jumping.

**TABLE 3:** Pre-test and post-test distribution of modified WeeFIM scores.

Modified WeeFIM	Mean	Median
Pre_WeeFIM	67.1	70.5
Post_WeeFIM	80.7	82.0

WeeFIM, Functional Independence Measure for Children.

### Normality test

In order to determine how the independent variable affects the dependent one, the Shapiro–Wilk test was utilised to first ascertain if the data distribution was normal. In light of the test’s outcome, the authors employed the Wilcoxon signed-rank test for data that were not normally distributed and the paired samples t-test for data that were normally distributed. The relationships between the variables were also investigated using the Pearson correlation test. For all inferential tests, the threshold for statistical significance was set at *p* < 0.05. The Wilcoxon signed-rank test, which was used to evaluate the GMFM-88 scores, revealed an increase in scores following the intervention, and are summarised in Table 4. Results demonstrated an increase in the mean scores across all five dimensions (A–E) following the intervention, suggesting improvement.

**TABLE 4:** Wilcoxon signed-rank test for GMFM-88 scores.

Variable	Mean ± s.d.	Asymp. Sig. (2-tailed)
Post_Pre DimA	4.750 ± 4.700	0.001
Post_Pre DimB	6.450 ± 6.245	0.001
Post_Pre DimC	4.950 ± 5.365	< 0.001
Post_Pre DimD	3.550 ± 4.273	0.002
Post_Pre DimE	3.350 ± 4.332	0.005

s.d., standard deviation; Asymp. Sig., asymptotic significance.

Note: Key for dimensions: Dim A: Lying and rolling, Dim B: Sitting, Dim C: Crawling and kneeling Dim D: Standing Dim E: Walking, running and jumping.

Additionally, a paired t-test was performed to compare the Modified WeeFIM scores both before and following the intervention. Table 5 displays the paired t-test outcomes for the Modified WeeFIM.

**TABLE 5:** Paired *t*-test for modified WeeFIM scores.

Post_Pre WeeFIM	Mean	s.d.	Sig. (2-tailed)
Variable	13 600	5134	< 0.001

s.d., standard deviation.

The analysis of the Modified WeeFIM scores revealed a significant improvement in the participants’ daily living abilities following the intervention (*p* < 0.001). The mean increase of 13.600 points indicates a substantial and clinically meaningful gain in functional independence. These findings strongly suggest that the NDT protocol was effective in enhancing the participants’ ability to carry out daily activities.

### The relationship between neurodevelopmental therapy administration and gross motor skills and activities of daily living

To assess the connection between NDT intervention and progress in motor abilities and daily functioning, a Pearson correlation analysis was conducted between total GMFM-88 and Modified WeeFIM scores. These findings are summarised in Table 6, which presents the detailed results of the Pearson correlation analysis.

**TABLE 6:** Correlation between GMFM-88 and modified WeeFIM post-intervention scores.

Variable	Sub-variable	Post_WeeFIM	Post_GMFM
Post_WeeFIM mean (s.d.)	Correlation coefficient	1.000	0.702
	Sig. (2-tailed)	-	< 0.001
	*n*	20.000	20.000
Post_GMFM median (IQR)	Correlation coefficient	0.702	1.000
	Sig. (2-tailed)	< 0.001	-
	*n*	20.000	20.000

WeeFIM, Functional Independence Measure for Children; GMFM, Gross Motor Function Measure; s.d., standard deviation.

Table 6 presents the results of paired t-tests comparing pre- and post-intervention scores. Significant improvements were observed in GMFM-88 and WeeFIM scores (*p* < 0.05), indicating that NDT positively impacted both gross motor function and daily living activities. The analysis showed a noteworthy positive correlation (*p* < 0.001, *r* = 0.702), suggesting that improvements in motor function are closely associated with increased independence in daily tasks.

## Discussion

This study demonstrated that an 8-week NDT intervention yielded statistically notable gains in children with spastic diplegia in terms of their independence in everyday activities and gross motor skills. The findings suggest that a structured NDT programme can be an effective modality for this specific population (Ahmed et al. 2025; Khanna et al. 2023).

The GMFM-88’s measurement of gross motor function improvement aligns with existing evidence. The results of this investigation are similar to the findings of Iftikhar et al.’s study 2024, which reported significant GMFM-88 improvements in children with spastic diplegia in Pakistan after an 8-week NDT intervention. Other studies, such as Labaf et al. (2015) and Tsorlakis et al. (2004), also demonstrated that NDT could significantly improve gross motor outcomes in children with CP, strengthening the consistency of our findings. The positive outcome in this study can likely be attributed to NDT’s emphasis on inhibiting atypical tone and facilitating postural control, which directly addresses the core motor impairments of spastic diplegia, particularly trunk weakness and challenges in motor planning (MacWilliams et al. 2022; Zahra et al. 2024).

Similarly, the enhanced independence in daily activities, reflected by a mean increase of 13.6 points on the Modified WeeFIM, highlights the functional impact of the therapy. This result reinforces Tekin et al.’s (2018) findings, which showed comparable gains in WeeFIM scores among children with spastic diplegia and hemiplegia in Turkey, using a similar intervention frequency. Given that the upper limb function of children with spastic diplegia is often less affected, the improvements in activities of daily living (ADL) may stem primarily from enhanced postural stability and mobility, allowing for greater independence in tasks such as dressing and transferring. Similarly, Park and Kim (2018) and Chokshi et al. (2021) also reported that higher therapy frequency and improved postural control were positively correlated with better ADL performance, supporting our finding (Kim et al. 2022).

Furthermore, this study found a strong, favourable relationship between enhancements in freedom in daily tasks and gross motor function (*r* = 0.702, *p* < 0.001). This finding empirically supports the ICF that posits that improvements in body function (e.g. motor skills) can lead to increased activity and participation in daily life (Emara 2015).

Despite the positive findings of this study, it is crucial to acknowledge the ongoing scientific debate surrounding NDT’s efficacy. A meta-analysis by Te Velde et al. (2022) concluded that NDT may have limited superiority over other task-specific interventions. Similarly, Novak and Damiano (2024) argue that although NDT remains widely used, stronger empirical validation is needed compared with more evidence-based, activity-focused approaches. Zanon (2018), in a Cochrane review, also emphasised the need for higher-quality trials to establish NDT’s clinical effectiveness. Therefore, while this study supports the use of NDT for specific goals in a targeted population, clinicians should consider integrating its principles with other evidence-based approaches to optimise outcomes for children who have CP.

In conclusion, the findings suggest that NDT is a valuable intervention for enhancing motor skills and functional autonomy in everyday tasks for children with spastic diplegia.

### Limitations

It is crucial to consider this study’s several limitations. Firstly, the pre-post experimental design lacked a control group, making it difficult to attribute the observed improvements solely to the NDT intervention. Secondly, generalisability is constrained by the tiny sample size as well as the fact that it was drawn from one private paediatric rehabilitation centre in an urban context in Indonesia; these results may not be applicable to children in public or rural healthcare settings. Thirdly, the study focused exclusively on children with spastic diplegia, which restricts the applicability of the findings to other forms of CP. The brief 8-week duration of the intervention made it difficult to observe any long-term clinical changes. The pre- and post-intervention tests were not done by independent researchers but by the same therapists who undertook the intervention.

## Conclusion

The findings from this small-sample study suggest that an 8-week NDT intervention was associated with improved gross motor abilities and daily task independence in children with spastic diplegia. Within this specific context, significant progress was observed in GMFM-88 and Modified WeeFIM scores. While these findings are positive, the study’s shortcomings, such as the lack of a reference group, the limited sample size and the lack of independent measurements, mean that any possible advantages of NDT in improving motor development and everyday functioning must be viewed cautiously.
